# Regional hyperthermia combined with chemotherapy in paediatric, adolescent and young adult patients: current and future perspectives

**DOI:** 10.1186/s13014-016-0639-1

**Published:** 2016-04-30

**Authors:** Georg Seifert, Volker Budach, Ulrich Keilholz, Peter Wust, Angelika Eggert, Pirus Ghadjar

**Affiliations:** Department of Paediatric Oncology and Haematology, Otto-Heubner Centrum für Kinder- und Jugendmedizin, Charité - Universitätsmedizin Berlin, Augustenburger Platz 1, 13353 Berlin, Germany; Department of Radiation Oncology, Charité - Universitätsmedizin Berlin, Augustenburger Platz 1, 13353 Berlin, Germany; Comprehensive Cancer Center, Charité - Universitätsmedizin Berlin, Virchowweg 23, 10115 Berlin, Germany

## Abstract

Here we evaluate the current status of clinical research on regional hyperthermia (RHT) in combination with chemotherapy or radiation therapy in paediatric oncology.

Data were identified in searches of MEDLINE, Current Contents, PubMed, and references from relevant articles using medical subject headings including hyperthermia, cancer, paediatric oncology, children, radiation therapy and chemotherapy. Currently, only two RHT centres exist in Europe which treat children. Clinical RHT research in paediatric oncology has as yet been limited to children with sarcomas and germ cell tumours that respond poorly to or recur after chemotherapy. RHT is a safe and effective treatment delivering local thermic effects, which may also stimulate immunological processes via heat-shock protein reactions. RHT is used chiefly in children and adolescents with sarcomas or germ cell tumours located in the abdomino-pelvic region, chest wall or extremities to improve operability or render the tumour operable. It could potentially be combined with radiation therapy in a post-operative R1 setting where more radical surgery is not possible or combined with chemotherapy instead of radiation therapy in cases where the necessary radiation dose is impossible to achieve or would have mutilating consequences. RHT might also be an option for chemotherapy intensification in the neoadjuvant first-line treatment setting for children and adolescents, as was recently reflected in the promising long-term outcome data in adults with high-risk soft tissue sarcomas (EORTC 62961/ESHO trial).

The limited data available indicate that combining RHT with chemotherapy is a promising option to treat germ cell tumours and, potentially, sarcomas. RHT may also be beneficial in first-line therapy in children, adolescents and young adults. The research should focus on optimising necessary technical demands and then initiate several clinical trials incorporating RHT into interdisciplinary treatment of children, adolescents and young adults that include translational research components exploring potential immunological mechanisms of action.

## Introduction – current situation

In contrast to adult oncology, tumours of mesenchymal and neuroectodermal origin predominate in paediatric oncology. While sarcomas occur in all age groups, they are relatively more common in children and adolescents than later in life. In the course of the years, co-operative treatment optimisation studies have led to marked optimisation of systemic polychemotherapy, for example for the group of soft tissue sarcomas [1]. The possibilities for optimisation of these conventional chemotherapy protocols appears to have reached a level where only new mechanisms of action and innovative strategies can produce further improvements with clinical relevance. Epigenetic and immunological treatment approaches can be expected to play an increasing role in the coming years, but these are still at the stage of clinical development. Successful treatment for sarcomas incorporates both effective systemic neo-/adjuvant therapy and local control even when the tumour has metastasised to improve overall survival (OS) [2]. Despite a frequently unfavourable location of the primary tumour, the aim is always to permit R0 resection with as little functional loss as possible. Here there have also been marked improvements in both surgical and radiation therapy strategies in the past years and new techniques have been introduced. In spite of modern radiation therapy techniques such as proton therapy, stereotactic radiation therapy or radiosurgery, and experimental procedures such as isolated limb perfusion chemotherapy, effective local therapy can present a success-limiting problem in the individual case. But even in the case of effective local therapy, acute and long-term side effects of surgery and/or radiation therapy can present a relevant problem.

Regional hyperthermia (RHT), offers an interesting approach that acts synergistically with chemotherapy and radiation therapy without producing disproportionate additional side effects, provided that current quality assurance guidelines for treatment planning and application are followed [3–5]. The rationale is a synergistic thermic chemosensitation through the use of alkylating substances such as cyclophosphamide or ifosfamide, platinum derivatives or anthracyclines [6] as established in the Hyper-PEI protocol and the german study group for malignant germ cell tumors (MAKEI) [7]. RHT in combination with chemotherapy is the best investigated thermotherapy procedure in both adult and paediatric oncology to date [8, 9]. This review aims to summarize the current knowledge on RHT in the treatment of paediatric, adolescent and young adult patients and provide some discussion points and future perspectives.

## Methods

Data for this paper were identified by searches of MEDLINE, Current Contents, PubMed, and references from relevant articles using medical subject headings including hyperthermia, cancer, paediatric oncology, children, radiotherapy and chemotherapy.

## Results

### Regional hyperthermia with chemotherapy

The search strategy identified six reports on prospective clinical trials without control groups (Table 1). The data on RHT or hyperthermic chemotherapy in children and adolescents are currently limited, and only two active German hyperthermia centres treating paediatric patients were identified, the Heinrich Heine University Hospital Düsseldorf and the Charité-Universitätsmedizin Berlin. The study by Romanowski et al. was the first which combined RHT and systemic chemotherapy (ICE: etoposide 4 × 150 mg/m^2^ and ifosfamide 4 × 2000 mg/m^2^ or carboplatin 4 x 150 mg/m^2^) in 34 intensively pretreated children and adolescents with prognostically unfavourable tumour diseases [10]. Romanowski et al. reported that 20 % of patients experienced a complete remission and 7 patients remained alive up to 64 months after the combined treatment. A second pilot trial treated 9 patients with locoregional recurrences of abdominal germ cell tumors with PEI chemotherapy (PEI: cisplatin 40 mg/m^2^ on days 1 and 4, etoposide 100 mg/m^2^ on days 1 to 4 and ifosfamide 2000 mg/m^2^ on days 1 to 4) in combination with RHT but with or without radiation treatment. Event-free survival (EFS) in these 9 patients (EFS 0.41 ± 0.33) who received RHT was significantly better than in a matched cohort (EFS 0.16 ± 0.25) who did not receive RHT, supporting the idea that RHT can have a relevant impact on local tumor control and patient EFS [11]. A further report on 10 patients from a phase I/II trial in children and adolescents with recurrent or refractory germ cell tumours treated at the University Hospital Düsseldorf with RHT combined with platinum-based PEI chemotherapy demonstrated feasibility and efficacy with objective tumour response rates up to 70 % [12]. Of these 10 patients, 2 responded partially and 5 had a complete response. Schneider et al. reported on a prospective trial without a control group for patients with relapsed malignant sacrococcygeal germ cell tumours who received cisplatin-based first-line polychemotherapy and tumour resection according to the MAKEI trial [7]. In this trial, 22 patients with first relapses, 14 with second relapses and 7 with later relapses were treated by surgery and adjuvant platinum chemotherapy. Pre-operative chemotherapy was administered together with RHT with the aim to improve operability in a subgroup of 8 poor responders or patients with locally advanced tumours. Although the small number of patients limits the informative value, the effect of RHT in this study was better when applied early resulting in 4 complete remissions after first or second relapses.

**Table 1:** Design of clinical studies employing regional hyperthermia with chemotherapy

Reference	Indication	Research design	Schedule of Hyperthermia/Number of sessions	Chemotherapy/Number of courses	Number of patients	Outcome measure	Comment
Romanowski Klinische Pädiatrie 1993 [10]	Different solid tumors with prognostically unfavorable tumor diseases	Feasibility of regional hyperthermia with systemic chemotherapy	Day 1 and 4 simultaneously to the chemotherapy (2–16 RHT sessions per patient; mean 6,5)	16 patients: IE: etoposide 4 x 150 mg/m^2^ and ifosfamide 4 x 2000 mg/m^2^ 8 patients: ICE: etoposide 4 x 150 mg/m^2^ and ifosfamide 4 x 2000 mg/m^2^ or every 2nd cycle carboplatinum 4 x 150 mg/m^2^ 10 patients received various substances including adriamycin Mean 3 treatment courses	34 (age: mean 11 years: 6 month)	Feasability study	First study in children and adolescents on RHT and Chemotherapy 20 % complete remission 7 patients were in CR up to 64 month
Wessalowski R. Klinische Pädiatrie 1997 [11]	Locoregional Recurrences of abdominal Germ Cell Tumors	Pilot study PEI-chemotherapy and RHT compared to a mached cohort	Day 1 and 4 simultaneously to the chemotherapy (6–30 RHT sessions per patient; mean 12,4)	PEI: cisplatinum 40 mg/m^2^ on day 1 and 4; etoposide 100 mg/m^2^ on day 1 to 4 and ifosfamide 2000 mg/m^2^ on day 1 to 4) Mean 6 treatment courses	9 RHT (age: mean 10 years; 11 month) 23 matched cohort	RHT subgroup 5-year EFS 0.41 ± 0.33 (5 CR, 2PR, 1SD, 1PD) matched cohort 5-year EFS 0.16 ± 0.25	
Wessalowski R. Cancer. 1998 [12]	Recurrence of an Malignant sacrococcygeal germ cell tumors	Phase I/II study	Day 1 and 4 simultaneously to the chemotherapy (6–10 RHT sessions per patient; mean 8,8)	PEI: cisplatinum 40 mg/m^2^ on day 1 and 4; etoposide 100 mg/m^2^ on day 1 to 4 and ifosfamide 2000 mg/m^2^ on day 1 to 4) Mean 4 treatment courses	10 (age: mean 11 years; 3 month)	Phase I/II study Toxicity (5 CR, 2PR, 1SD. 1NC, 1PD)	WHO grade 4 neutropenia and thrombocytopenia after all cycles Granulocytopenic fever after 27 % cycles; WHO grade-3 acute renal toxicity after 5 % cycles; WHO grade 3 mucositis after 11 % cycles
Schneider D. J Clin Oncol, 2001 [7]	Relapsed or refractory germ cell tumour	Subgroup in a prospective study no control group	Day 1 and 4 simultaneously to the chemotherapy	PEI: cisplatinum 40 mg/m^2^ on day 1 and 4; etoposide 100 mg/m^2^ on day 1 to 4 and ifosfamide 2000 mg/m^2^ on day 1 to 4)	A subgroup of 8 Patients (age: mean 11 years; 3 month) with RHT out of 22 first-, 14 s-, 5 third-, and 2 fourth-relapse	5 CR, 2 PR	
Wessalowski R. Klinische Pädiatrie 2003 [9]	Unresectable malignant tumours (24 extracranial non-testicular germ cell tumours; 15 soft tissue or chondrosarcomas)	Phase-II study PEI-chemotherapy and RHT	Day 1 and 4 simultaneously to the chemotherapy (6–10 RHT sessions per patient; mean 8,5)	PEI: cisplatinum 40 mg/m^2^ on day 1 and 4; etoposide 100 mg/m^2^ on day 1 to 4 and ifosfamide 2000 mg/m^2^ on day 1 to 4) Three to four treatment courses	24 Germ cell tumours (age: mean 3 years; 7 month) 15 Soft tissue sarcoma (age: mean 7 years; 6 month)	Whole study cohort median follow-up 27 months 5-year EFS 0.39 +/− 0.11 (20/39 patients) 5-year OS 0.52 +/− 0.11 (25/39 patients)	13 patients received local radiotherapy
Wessalowski R. The Lancet Oncology, 2013 [8]	Relapsed or refractory germ cell tumour	Prospective study no control group	Day 1 and 4 simultaneously to the chemotherapy (6–10 RHT sessions per patient; mean 8,2)	PEI: cisplatinum 40 mg/m^2^ on day 1 and 4; etoposide 100 mg/m^2^ on day 1 to 4 and ifosfamide 2000 mg/m^2^ on day 1 to 4) Three to four treatment courses	44 (age: median 2 years; 7 months)	response to treatment 16 patients had CR and 14 had PR median follow-up 82 months 5-year event-free survival was 62 % (95 % CI 45–75) 5-year overall survival was 72 % (95 % CI 55–83).	Local radiotherapy if incompletely resected WHO grade 3–4 neutropenia and thrombocytopenia after every cycle. Granulocytopenic fever in 29 (66 %) of 44 patients after 53 (29 %) cycles. WHO grade-3 acute renal toxic effects in five patients

The group of Wessalowski at the University Hospital Düsseldorf published two further trials. The first trial included 39 patients, 24 with extracranial non-testicular germ cell tumours and 15 with soft tissue sarcomas or chondrosarcomas, of which 29 tumours were locoregional relapses and 10 were unresectable following pre-operative chemotherapy. Of these 39 patients treated with RHT according to the Hyper-PEI protocol, 20 achieved complete remission and 10 achieved partial remission. A consecutive surgical tumour resection was possible in 28 of these patients, while 13 patients required additional radiotherapy. After 5 years of follow-up, EFS was 0.39 ± 0.11 (20/39 patients) and OS was 0.52 ± 0.11 (25/39 patients). This interdisciplinary treatment of RHT with chemotherapy enabled a successful local therapy leading to a sustained remission in most patients with high risk for tumour recurrence [9]. Wessalowski et al. also used RHT in combination with chemotherapy for salvage treatment in 44 children and adolescents with refractory or recurrent non-testicular malignant germ-cell tumours [8]. Sufficient data could only be obtained for 35 of the 44 patients, of which 30 (86 %) had an objective response to the combination of RHT and PEI chemotherapy. PEI-regional deep hyperthermia utilised 40 mg/m(2) cisplatin delivered intravenously on days 1 and 4, 100 mg/m(2) etoposide delivered intravenously on days 1–4 and 1800 mg/m(2) ifosfamide delivered intravenously on days 1–4. One hour of RHT (41–43 °C) on days 1 and 4 was applied simultaneously with chemotherapy. Wessalowski et al. showed that 16 of these patients achieved complete remission and 14 achieved partial remission. The 5-year EFS and OS was 62 and 72 %, respectively, and toxicity was tolerable with only reports of WHO grade 3–4 neutropenia and thrombocytopenia occurring after all chemotherapy cycles. This was the first data showing that RHT combined with PEI could improve the long-term outcome of children and adolescents with refractory or recurrent malignant non-testicular germ-cell tumours.

Notably, more recent data from adult patients with high-risk soft tissue sarcomas treated within the EORTC 62961/ESHO randomised trial showed that peri-operative chemotherapy combined with RHT significantly improved either disease-free survival or OS [13, 14], supporting combined RHT and chemotherapy as a reference therapy for adults with high-risk soft tissue sarcomas. These results indicated that RHT not only improved the local chemotherapy effect, but that it may also have stimulated immunological processes which could permit longer-term tumour control [15, 16]. Whether this approach would also be successful in first-line treatment of paediatric patients with sarcomas has yet to be demonstrated.

### Further hyperthermia procedures

There are no data from trials combining RHT and radiation therapy (thermoradiotherapy) in paediatric patients, however, the limited experience reported in case reports on paediatric patients support that application would probably have similar synergistic effects as in adults. Results from the combination of radiation therapy and hyperthermia for locally advanced cervical cancer in adult patients are worth mentioning in this context. The Dutch Deep Hyperthermia Trial not only showed an 83 % local control rate for combined radiation therapy and RHT versus 57 % for radiation therapy alone, but also a 3-year OS of 51 % for radiation therapy combined with RHT, compared to 27 % with radiation therapy alone [17]. The 12-year OS was still almost twice as high after combined radiation therapy and RHT (37 %), versus 20 % with radiation therapy alone [18].

There is currently no data available for whether the post-operative use of RHT together with radiation therapy in on paediatric patients is beneficial compared to radiation therapy alone. It remains unclear whether radiation therapy could be omitted under certain circumstances and the R1 area treated instead with a combination of RHT and chemotherapy. Future prospective trials are necessary to evaluate the outcome and toxicities of these approaches [18].

### Whole-body hyperthermia

Less is known regarding whole-body hyperthermia in children, a procedure aiming to establish a steady state with maximal temperatures of 42 °C that is maintained for 1 h. Such a procedure can be achieved only with deep analgesia and sedation or general anaesthesia using radiant systems which have been reviewed before [19]. Therefore, the efforts needed (including intensive medical care) are much greater than for locoregional methods. Apart from a few individual cases [19–21], there is practically no experience with whole-body hyperthermia in children. There is also no solid data available on the use of whole-body hyperthermia in adults [19].

### Special indications such as desmoid tumours/aggressive fibromatosis

There were a number of positive reports on RHT and its combination with radiation therapy in patients with desmoid tumours. To date, some limited data for individual cases in paediatric patients support that it could be an appropriate second-line option for complex refractory situations [22–25].

## Discussion

### Technical solutions for RHT in children

In paediatrics, body weights range from under 5 kg to adult weight. This means that the technical requirements for the widely differing weights and, thus, widely differing body and organ dimensions are complex. In addition to the special body dimensions in children, their stress tolerance for RHT is considerably lower and decreases with the age of the child. Experience has shown that from the beginning of puberty children are able to tolerate an RHT session without deep sedation or general anaesthesia. Table 2 shows the dimensions of the commercially available applicators that can be used in children together with the respective patient and tumour dimensions treatable. These technical solutions can generate intratumoral temperatures of up to 43 °C, which correspond to the successful treatment protocols in children and adults.

**Table 2:** Available applicators for use in children and adolescents

Available applicators with dimensions, treatable craniocaudal tumour length and permitted maximal lateral and dorsoventral dimensions of the patient			
	Sigma 30	Sigma 40	Sigma Eye
Applicator length (bolus)	25	40	50
Applicator internal diameter (bolus)	27x27	42	54 x 37
Approximate maximal craniocaudal tumour length	~15–20	~25–30	~35–40
Maximal lateral (x) and dorsoventral (y) patient dimensions (pelvis/abdomen)	x ≤ 19 cm y ≤ 19 cm	19 cm < x ≤ 34 cm 19 cm < y ≤ 34 cm	19 cm < x ≤ 44 cm 19 cm < y ≤ 29 cm

### Feasibility in children and side effects

The same body regions accessible in adults are accessible in children, with bones, lungs and the CNS being difficult to reach in all patients regardless of age. Children do have some special needs during RHT, however, and require more analgesics and need them applied earlier in combination with deep sedation or anaesthesia during RHT application [11, 12]. There are no concerns against RHT in combination with deep sedations or narcosis, although the temperature around certain organs at risk, including the skin, must be monitored carefully because of the missing perception of pain. Side effects of RHT are temperature-associated and generally rare events, as children are considerably more tolerant to temperature elevation than adults. Genuine burns are very rare. The greater risk is associated with the additional chemotherapy or radiation therapy and their associated toxicities. RHT offers a possible method to intensify local therapy without significant additional toxicity.

## Conclusion

RHT can be considered for children with solid tumours, particularly germ cell tumours and soft-tissue sarcomas, and in cases of refractory tumours, recurrences or situations where standard treatments cannot be used. RHT should be applied early in the relapse situation. An important conclusion from the analysed trials is the observation that patients with recurrent germ cell tumours can be cured by RHT, even in cases where cispatin resistence arose during earlier therapy. RHT could be considered in individual cases with complex refractory situations for rare tumours, such as desmoid tumours. Currently available data from children [8, 12] and the more recent data from adults with soft tissue sarcomas [14] indicate that RHT could be an effective treatment option that should be further developed and investigated in children. Long-term immunological effects that potentially result from the heat-shock reaction should be closely monitored in children receiving RHT [26, 27], since clinical data to date suggest that the effects go beyond improved local control [14]. A prospective treatment optimisation trial for RHT combined with chemotherapy in the second-line treatment of sarcomas in children, adolescents and young adults is in preparation at the Charité – Universitätsmedizin Berlin.
